# Cost-effectiveness analysis of natural birth and elective C-section in supplemental health

**DOI:** 10.11606/S1518-8787.2018052000373

**Published:** 2018-11-14

**Authors:** Aline Piovezan Entringer, Márcia Pinto, Maria Auxiliadora de Souza Mendes Gomes

**Affiliations:** I Instituto Nacional de Saúde da Mulher, da Criança e do Adolescente Fernandes Figueira. Fundação Oswaldo Cruz. Neonatologia. Rio de Janeiro, RJ, Brasil; II Maternidade Escola da Universidade Federal do Rio de Janeiro. Unidade de Terapia Intensiva Neonatal. Rio de Janeiro, RJ, Brasil; III Instituto Nacional de Saúde da Mulher, da Criança e do Adolescente Fernandes Figueira. Fundação Oswaldo Cruz. Departamento de Pesquisa Clínica. Rio de Janeiro, RJ, Brasil; IV Instituto Nacional de Saúde da Mulher, da Criança e do Adolescente Fernandes Figueira. Fundação Oswaldo Cruz. Unidade de Pesquisa Clínica. Rio de Janeiro, RJ, Brasil

**Keywords:** Natural Childbirth, economics, Cesarean Section, economics, Cost-Effectiveness Evaluation, Supplemental Health, Parto Normal, economia, Cesárea, economia, Avaliação de custo-efetividade, Saúde Suplementar

## Abstract

**OBJECTIVE:**

To conduct a cost-effectiveness analysis of natural childbirth and elective C-section for normal risk pregnant women.

**METHODS:**

The study was conducted from the perspective of supplemental health, a health subsystem that finances private obstetric care, represented in Brazil by health plan operators. The reference populations were normal risk pregnant women, who could undergo natural childbirth or elective C-section, subdivided into primiparous and multiparous women with previous uterine scar. A decision analysis model was constructed including choice of delivery types and health consequences for mother and newborn, from admission for delivery to maternity hospital discharge. Effectiveness measures were identified from the scientific literature, and cost data obtained by consultation with health professionals, health plan operators’ pricing tables, and pricing reference publications of health resources.

**RESULTS:**

Natural childbirth was dominant compared with elective C-section for primiparous normal risk pregnant women, presenting lower cost (R$5,210.96 *versus* R$5,753.54) and better or equal effectiveness for all evaluated outcomes. For multiparous women with previous uterine scar, C-section presented lower cost (R$5,364.07) than natural childbirth (R$5,632.24), and better or equal effectiveness; therefore, C-section is more efficient for this population.

**CONCLUSIONS:**

It is necessary to control and audit C-sections without clinical indication, especially with regard to primiparous women, contributing to the management of perinatal care.

## RESUMO

**OBJETIVO:**

Realizar uma análise de custo-efetividade do parto vaginal e da cesariana eletiva para gestantes de risco habitual.

**MÉTODOS:**

A perspectiva adotada foi a da saúde suplementar, subsistema de saúde financiador da assistência obstétrica privada, representado no Brasil por operadoras de planos de saúde. As populações de referência foram as gestantes de risco habitual, que poderiam ser submetidas ao parto vaginal ou à cesariana eletiva, subdivididas em primíparas e multíparas com uma cicatriz uterina prévia. Foi construído um modelo de decisão analítico que incluiu a escolha pelos tipos de parto, consequências em saúde para mãe e recém-nascido da internação para o parto à alta da maternidade. As medidas de efetividade foram identificadas a partir da literatura científica. Os dados de custos foram obtidos pela consulta aos profissionais de saúde, tabelas das operadoras dos planos de saúde e publicações de referências de preços de recursos de saúde.

**RESULTADOS:**

O parto vaginal foi dominante em comparação com a cesariana eletiva para gestantes de risco habitual primíparas e apresentou menor custo (R$5.210,96 *versus* R$5.753,54) e melhor ou igual efetividade para todos os desfechos avaliados. Para multíparas com uma cicatriz uterina prévia, a cesariana mostrou-se com custo inferior (R$5.364,07) ao do parto vaginal (R$5.632,24) e melhor ou igual efetividade, portanto mais eficiente para essa população.

**CONCLUSÕES:**

É necessário o controle e a auditoria das cesarianas sem indicação clínica, destacadamente em primíparas, contribuindo para a gestão da atenção perinatal.

## INTRODUCTION

Brazil is among the countries with the highest rate of C-sections in excess ^1^ , and in 2014 it reached 57% in the country ^a^ . When disaggregated to the Unified Health System (SUS) and supplemental health care, this rate is around 43% and 85%, respectively ^2^ . The World Health Organization (WHO) indicates that rates above 10% are not associated with reduced maternal and neonatal mortality ^b^ . Currently in Brazil, due to a high rate of previous C-sections, population’s characteristics and obstetric model, the indicator reference suggested by the *Diretrizes de Atenção à Gestante: a operação Cesariana* (Guidelines for Caring the Pregnant Woman: C-section) varies between 25% and 30% ^c^ .

Brazilian population’s access to health care occurs by the Unified Health System (SUS), which is universal and financed exclusively with public resources; and by the segment of elective private health plans, financed with resources from families and/or employers, and out-of-pocket payments ^d^ . Supplemental health care in Brazil is regulated by the National Regulatory Agency for Private Health Insurances and Plans (ANS), responsible for regulating 789 active medical and hospital operators in 2016 ^e^ . In supplemental health care, remuneration is mostly performed by the fee-for-service model, which remunerates per unit of service, procedure packages and hospital daily rates. Although this model is more associated with the unnecessary increased care cost, it is the predominant way of financing in supplemental health care services ^d^ .

In 2015, health plan operators were responsible for the payment of almost 20% deliveries throughout the country ^f^ ^,^ ^g^ . In the same year, there were approximately 569,000 deliveries in the accredited health network, and 85% deliveries were by C-sections ^h^ .

Since 2004, ANS has implemented strategies for the reduction of C-sections in supplemental health care, via the Qualification Program ^i^ . In 2015, with the publication of Normative Resolution 368, ANS adopted a series of measures to improve obstetric practice, such as the inclusion of the pregnant woman’s card, the partograph, and a detailed description of the rate of C-sections per health care provider, establishment and doctor ^j^ . Moreover, the *Parto Adequado* (Proper Delivery) project was implemented aiming at testing strategies to improve delivery, change the current model and qualify the services ^k^ .

Surgical deliveries bring benefits to maternal and perinatal health when performed with clinical justification. However, longer hospital stay and higher maternal and neonatal morbidity may occur if women are submitted to the procedure without adequate indication^3 – 14^ .

Several aspects are related to choosing between the two types of delivery, such as pregnant women’s and professionals’ preference, favorable health outcomes, in addition to economic issues, due to the difference in cost between procedures. In this sense, economic evaluations in health contribute to supporting managers’ decision-making. Economic evaluation studies in this field are scarce in Brazil. Results generated by research of this nature may be added to the evidence of safety and clinical efficacy established in the literature. This study aimed at performing cost-effectiveness analysis of natural childbirth and elective C-section from supplemental health care perspective.

## METHODS

Cost-effectiveness analysis comparing natural childbirth and elective C-section without clinical indication, from the perspective of supplemental health care, a health subsystem financing private obstetric care that remunerates health services via health plan operators. The reference populations were normal risk pregnant women who could undergo both procedures. A normal risk pregnant woman is that with no clinical and obstetric complications until the moment of delivery, carrying a single full-term fetus (37 to 41 weeks of gestational age) with cephalic presentation. Pregnant women who could benefit from cesarean section were not included ^c^ . The population was subdivided into primiparous and multiparous women with previous uterine scar, in order to separately evaluate the incremental cost-effectiveness of natural childbirth after C-section and repeat C-section.

Natural childbirth was compared with elective C-section by intention to treat. The “natural childbirth” group referred to the women who planned the natural childbirth and went into labor spontaneously, but may have undergone intrapartum C-section. The natural childbirth considered was either spontaneous or in need of assistance. The “elective C-section” group was the comparator. The concept of elective C-section applies to that pregnant woman who undergoes a scheduled surgical intervention performed before labor begins, and the amniotic membranes are intact. In this study, the elective C-section considered was that without clinical justification. The time horizon comprised the period from admission for delivery to maternity hospital discharge.

**Figure 1:**
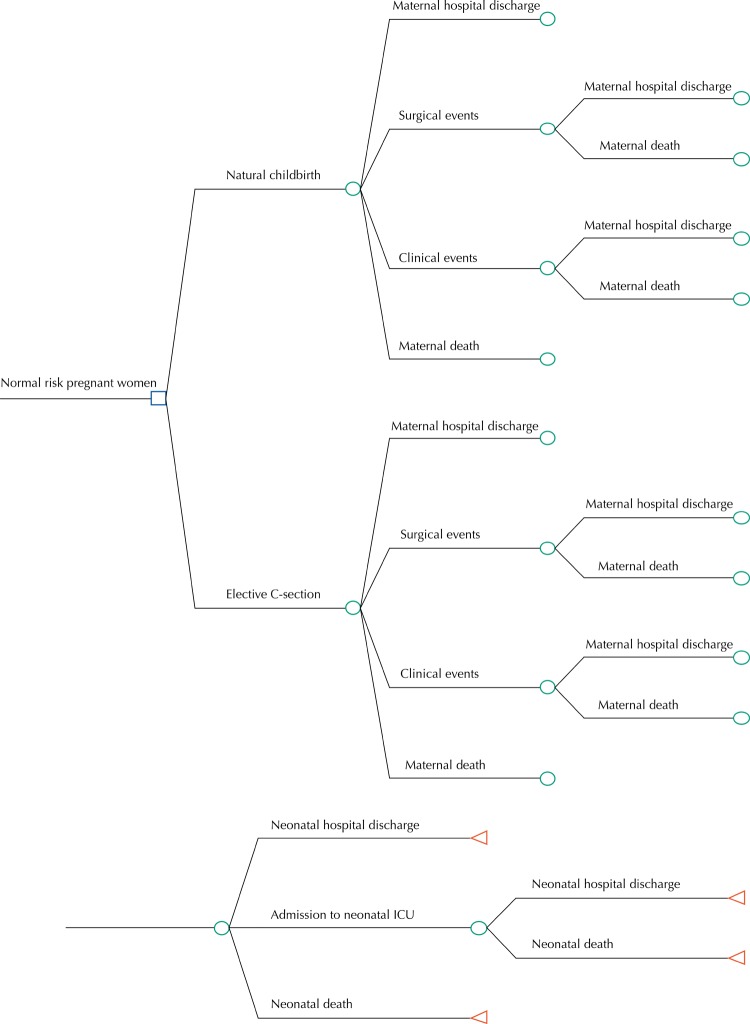
Analytical model of decision for normal risk pregnant women and newborns eligible for spontaneous natural childbirth, or C-section without clinical indications.

For the calculation of incremental cost-effectiveness ratio (ICER), an analytical decision model was constructed. This model was represented by a decision tree, which included the choice of delivery types, health consequences and interest final outcome for the mother and the newborn ( Figure 1 ). The decision model incorporated events that could occur during and after delivery, and were divided into maternal clinical events – hemorrhage requiring blood transfusion, and thrombosis/embolism – and maternal surgical events, such as hysterectomy, uterine rupture and maternal death. For the newborn, the study considered Neonatal Intensive Care Unit (NICU) stay and neonatal death. Effectiveness measures were obtained from the scientific literature, and Medline (via PubMed) and Scielo databases were used for bibliographic search. The search strategy used was [(“elective cesarean” OR “elective cesarean deliveries” OR “elective cesarean delivery” OR “vaginal delivery” OR “natural childbirth”) AND (“outcomes pregnancy” OR “maternal mortality” OR “neonatal mortality” OR “neonatal morbidity” OR “maternal morbidity” OR “preterm birth” OR “hemorrhage” OR “thrombosis” OR “respiratory stress” OR “urinary incontinence” OR “infection” OR “early term” OR “late preterm”)]. It encompassed articles published between 2000 and 2014, since aimed at identifying more recent information, consistent with the current clinical practice. The research selected articles including normal risk pregnant women, single fetus, and natural childbirth of fetus in cephalic presentation, which compared planned natural childbirth with elective C-section in relation to maternal and neonatal morbimortality. The articles which performed intention-to-treat analysis participated in the study.

ICU: Intensive Care Unit

The days of hospitalization were obtained from the Hospital Information System of the Unified Health System ^l^ (SIH-SUS), and from health professionals with more than 15 years of experience in obstetric and perinatal care, who worked in hospitals and clinics which are paid to render services to health plan operators ( Table 1 ).

**Table 1:** Variables of the decision model for primiparous and multiparous women and newborns.

Variable	Reference case	Variation (minimum-maximum)		Source
Primiparous women				

Natural childbirth (labor)				
Emergency C-section	0.113	0.082	0.154	^8,14,9^
Hemorrhage with blood transfusion	0.004	0.0032	0.019	^10,6,8^
Thrombosis/Embolism	0.003	-	-	^14^
Uterine rupture	0.00029	-	-	^14^
Hysterectomy	0.001	0.0001	0.002	^6,10,14^
Maternal death	0.0002	0	-	^14,10^
Admission to NICU	0.063	-	-	^3^
Neonatal death	0.0007	-	-	^4^
Elective C-section				
Hemorrhage with blood transfusion	0.003	0.0029	0.017	^8,10,6^
Thrombosis/Embolism	0.006	-	-	^14^
Uterine rupture	0.00015	-	-	^14^
Hysterectomy	0.006	0.001	0.006	^14,10,6^
Maternal death	0	0		^14.10^
Admission to NICU	0.139	-	-	^3^
Neonatal death	0.0017	-	-	^4^

Multiparous women				

Natural childbirth (labor)				
Emergency C-sector	0.26	0.08	0.28	^7.18^
Hemorrhage with blood transfusion	0.0066	0.002	0.022	^7^
Thrombosis/Embolism	0.04	-	-	^7^
Uterine rupture	0.0071	0.0052	0.0097	^7^
Hysterectomy	0.0014	0.0008	0.0022	^7^
Maternal death	0.000019	0.000004	0.000095	^7^
Admission to NICU	0.088	-	-	^5^
Neonatal death	0.0011	0.0006	0.002	^7^
Elective C-section				
Hemorrhage with blood transfusion needed	0.0046	0.0016	0.013	^7^
Thrombosis/Embolism	0.1	-	-	^7^
Uterine rupture	0.0002	0.00003	0.0018	^7^
Hysterectomy	0.0016	0.0007	0.0036	^7^
Maternal death	0.000096	0.000021	0.0004	^7^
Admission to NICU	0.083	-	-	^5^
Neonatal death	0.0006	0.0002	0.0015	^7^

Hospital stay days				

Shared room after C-section	2.6	2	4	^a,b^
Shared room after natural childbirth	2.1	1	3	^a,b^
Maternal ICU after clinical event	2	1	5	^b^
Maternal ICU after surgical event	3	2	5	^b^
Neonatal ICU	3	2	5	^b^
Shared room after clinical event	4	3	5	^b^
Shared room after surgical event	4	3	5	^b^
Days before maternal or neonatal death	5	2	10	^b^

NICU: Neonatal Intensive Care Unit; ICU: Intensive Care Unit

^a^ Ministério da Saúde (BR), Departamento de Informática do SUS (DATASUS). Internações hospitalares no SUS - por local de internação - Brasil. Brasília (DF); s.d. [cited 2014 Feb 3]. Available from: http://tabnet.datasus.gov.br/cgi/deftohtm.exe?sih/cnv/sxuf.def

^b^ Consultation with health professionals.

Direct costs of the procedures, events and outcomes were estimated for the following cost items: medicines, hospital supplies, hospital stay and medical fees.

The Brazilian Hierarchical Classification of Medical Procedures (CBHPM) of 2016 was used for determination of medical fees ^m^ . The CBHPM does not present either operating room rates or hospital daily rates; this information was obtained from national health plan operators’ pricing tables. The operators selected provided Information on hospital daily rates and operating room rates of the Southeast region and Federal District. For elective C-section and intrapartum C-section, there was inclusion of medical fees for one obstetrician, one assistant, anesthesia procedure and one pediatrician attending in the delivery room. Regarding natural childbirth, there was inclusion of medical fees for one obstetrician, one pediatrician attending in the delivery room, and anesthesia procedure for 31.5% natural childbirths, according to information from “Birth in Brazil,” a study which identified this percentage as hospital practice in the country ^15^ . There was addition of six hours of obstetric labor assistance for natural childbirth and intrapartum C-section. With regard to adult and neonatal ICU, on-call service and employee daily fees were considered. In surgical events, the medical fees corresponded to one surgeon, two assistants, and anesthesia procedure; for clinical events, there was inclusion of the suggested fee for blood transfusion procedure.

Identification and quantification of supplies and medicines used in the procedures and during the hospital stay after delivery, in apartment or neonatal or adult ICU, were also obtained via consultation with health professionals, and classified both from Simpro Hospitalar Magazine and from the Brasíndice Pharmaceutical Guide, used for negotiation between health service providers and health care operators in Brazil. For medicines, the study considered the factory price ^n^ from the Brasíndice Pharmaceutical Guide, and the 17.5% tax on Circulation of Goods and Services (ICMS) was applied for the reference case. For hospital supplies, a 30% additional amount from Simpro Hospitalar Magazine was considered. For natural childbirth, the calculation of hospital supplies cost was based on data from the study “Birth in Brazil” for normal risk pregnant women who went into labor. These data indicated 73.8% pregnant women had venipuncture; 38.2% used oxytocin; 31.5% required analgesia, and 56% had episiotomy ^15^ . These percentages were multiplied by the cost of hospital supplies.

The data collection from specialists was carried out in person and individually with each of them. An script was used for each unit included (obstetric center, surgical center, shared room, neonatal and adult intensive care unit) to identify all supplies and medicines used from admission for delivery (first delivery period for natural childbirth, and admission to the surgical center for elective C-section); procedure conduction; postpartum period; newborn assistance in the delivery room and mother stay; baby and companion in shared room, and days of stay in each unity. The selected professionals were nurses, physicians, pediatricians and anesthesiologists who work in reference services in Rio de Janeiro, such as hospitals and clinics that are paid to render services to health plan operators, with experience of more than 15 years in obstetric and perinatal care and availability to participate in the study. The professionals answered only the questionnaire referring to their specialty.

No discounts and inflation adjustments were applied due to the short-time horizon. The results were presented in 2016 Reais (R$).

The ICER, that is, the incremental cost per unit of benefit obtained, was calculated for the two populations studied, for the following outcomes: maternal death avoided; neonatal death avoided; maternal morbidity avoided, and neonatal morbidity avoided. For natural childbirth after a C-section and repeat C-section, the ICER was calculated for uterine rupture avoided. The cost-effectiveness threshold considered was of 71% Gross Domestic Product (GDP) per capita (R$26,000), as proposed in the international literature for middle income countries ^16^ and not formally established in Brazil.

Economic evaluations may include parameters that generate uncertainties. Thus, a deterministic and probabilistic sensitivity analysis was performed using the Monte Carlo microsimulation method, with 10,000 interactions, triangular distribution for costs and beta distribution for the probabilities. Effectiveness parameters were varied from the literature data ( Table 1 ). Medical fees, room rates and hospital daily rates were varied with decrease and increase of 20% reference case. Hospital supplies were varied using the values presented in Simpro Hospitalar Magazine with up to 50% increase. Hospital medicines were varied from 12% to 20% ICMS based on the Brasíndice Pharmaceutical Guide ( Table 2 ). The maximum cost variation of natural childbirth considered the anesthetic procedure for all pregnant women.

**Table 2:** Procedures costs of spontaneous natural childbirth, elective C-section and clinical and surgical events. Rio de Janeiro, State of Rio de Janeiro, 2016.

Variable	Reference case		Variation	
	
	R$	%	Minimum	Maximum
Natural childbirth				
Medical fees	2,727.63	78	2,182.10	3,727.32
Supplies and medicines	254.83	7	202.64	384.75
Room rate	526.40	15	421.12	631.68
Total	3,508.85	100	2,805.86	4,743.76
C-section				
Medical fees	2,123.81	62	1,699.05	2,548.57
Supplies and medicines	723.06	21	581.17	812.95
Surgical and post-anesthetic room rate	582.40	17	465.92	698.88
Total	3,429.27	100	2,746.13	4,060.40
Intrapartum C-section				
Medical fees	3,425.33	72	2,740.26	4,110.39
Supplies and medicines	760.63	16	610.79	856.36
Surgical and post-anesthetic room rate	582.40	12	465.92	698.88
Total	4,768.36	100	3,816.98	5,665.64
Mother/baby apartment				
Medical fees	72.31	13	57.85	86.77
Supplies and medicines	104.14	19	80.80	119.67
Apartment daily fee	368.20	68	294.56	441.84
Total	544.65	100	433.20	648.28
Neonatal ICU				
Medical fees	525.49	29	420.39	630.59
Supplies and medicines	353.00	19	229.95	332.56
Daily rate	951.00	52	760.80	1,141.20
Total	1,829.49	100	1,411.15	2,104.35
Adult ICU				
Medical fees	525.49	27	420.39	630.59
Supplies and medicines	608.43	31	523.20	705.11
Daily rate	827.54	42	662.03	993.05
Total	1,961.46	100	1,605.62	2,328.75
Surgical event				
Medical fees	2,300.29	59	1,840.23	2,760.35
Supplies and medicines	825.38	21	859.75	1,215.74
Room rate	789.69	20	631.75	947.63
Total	3,915.36	100	3,331.74	4,923.71
Clinical event				
Medical fees	343.70	66	274.96	412.44
Supplies and medicines	175.80	34	143.99	199.72
Total	519.50	100	418.95	612.16

ICU: Intensive Care Unit

Tree Age Pro 2015 ^17^ software was used to construct the decision model and calculate the ICER.

The study was approved by the Research Ethics Committee (Protocol 44387715.1.0000.5269).

## RESULTS

The direct cost of natural childbirth was R$3,508.85. Professional fees were the main cost drivers (78%), followed by room rates (15%). The elective C-section presented lower cost (R$3,429.27) than natural childbirth. Professional fees were also the most representative cost item (62%), followed by hospital supplies (21%) ( Table 2 ).

Surgical events presented cost of R$3,915.36, where 59% were for medical fees. In turn, the cost of clinical events was R$519.50, and 66% were for medical fees, and 34% for hospital supplies ( Table 2 ).

The total daily cost for the mother/newborn apartment was R$544.65, and 68% corresponded to the hospital daily rate. The total daily cost for adult ICU was R$1,962.46, where the hospital daily rate was also the main cost driver, representing 42%. The total daily cost for the neonatal ICU was R$1,829.49, and 52% corresponded to the hospital daily rate ( Table 2 ).

The analytical decision model, which included clinical events at delivery and birth of both procedures for mother and newborn, identified that the total cost of elective C-section (R$5,753.54) was higher than that of natural childbirth (R$5,210.96). Natural childbirth presented better effectiveness for the outcomes maternal morbidity avoided and admission to neonatal ICU avoided, and equal effectiveness for maternal death avoided and neonatal death avoided. Thus, it was dominant for primiparous normal risk pregnant women for all outcomes evaluated ( Table 3 ).

**Table 3:** Incremental cost-effectiveness analysis of natural childbirth and elective C-section for normal risk primiparous and multiparous pregnant women. Rio de Janeiro, State of Rio de Janeiro, 2016.

Variable	Total cost (R$)	Additional cost (R$)	Effectiveness	Incremental effectiveness	Incremental cost-effectiveness ratio
Primiparous women					

Outcome: Maternal death avoided					
C-section	5,753.54	524.58	0.98	-	Dominated
Natural childbirth	5,210.96	-	0.99	0.01	Dominant
Outcome: maternal death avoided					
C-section	5,753.54	524.58	1	-	Dominated
Natural childbirth	5,210.96	-	1	-	Dominant
Outcome: admission to NICU avoided					
C-section	5,753.54	524.58	0.86		Dominated
Natural childbirth	5,210.96	-	0.94	0.08	Dominant
Outcome: neonatal death avoided					
C-section	5,753.54	524.58	1	-	Dominated
Natural childbirth	5,210.96	-	1	-	Dominant

Multiparous women (one previous scar)					

Outcome: maternal death avoided					
C-section	5,364.07		0.99	0.01	Dominant
Natural childbirth	5,632.24	268.17	0.98		Dominated
Outcome: neonatal death avoided					
C-section	5,364.07		1		Dominant
Natural childbirth	5,632.24	268.17	1		Dominated
Outcome: uterine rupture avoided					
C-section	5,364.07		1	0.01	Dominant
Natural childbirth	5,632.24	268.17	0.99		Dominated
Outcome: admission to NICU avoided					
C-section	5,364.07		0.92	0.01	Dominant
Natural childbirth	5,632.24	268.17	0.91		Dominated
Outcome: neonatal death avoided					
C-section	5,364.07		1		Dominant
Natural childbirth	5,632.24	268.17	1		Dominated

NICU: Neonatal Intensive Care Unit

**Figure 2:**
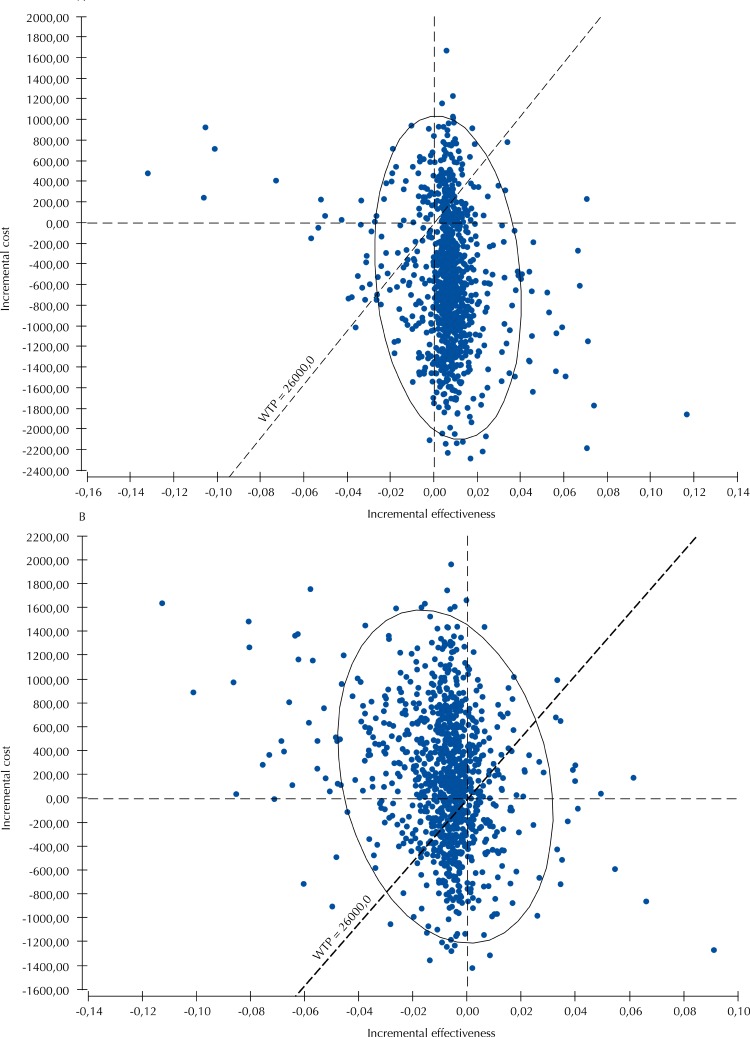
Probabilistic sensitivity analysis of natural childbirth and elective C-section for maternal morbidity avoided, for primiparous (A) and multiparous (B) women. Rio de Janeiro, state of Rio de Janeiro, 2016.

In the probabilistic sensitivity analysis ( Figure 2 ), natural childbirth presented 83% chance of being cost-effective for maternal morbidity avoided, 80% for maternal death avoided, 82% for neonatal death avoided, and 99% for admission to neonatal ICU avoided.

WTP: Willingness to pay

For multiparous pregnant women with previous scar, the analytical decision model identified a higher cost for natural childbirth (R$5,632.24) compared with elective C-section (R$5,364.07). Effectiveness was the same for maternal death avoided and neonatal death avoided, and higher for C-section regarding maternal morbidity avoided, uterine rupture avoided, and admission to neonatal ICUs avoided. Elective C-section was the most cost-effective option for multiparous women with previous uterine scar ( Table 3 ).

The variables that most affected the cost-effectiveness model of multiparous pregnant women on Tornado diagram were the following: cost of elective C-section; days of stay in shared room for elective C-section, and the probability of intrapartum C-section in the natural childbirth group.

There was consideration of the interval of 8% ^18^ to 28% ^7^ probability of occurrence of intrapartum C-section in the natural childbirth group in the univariate sensitivity analysis of the parameter “probability of intrapartum C-section in the natural childbirth group”. In this analysis, natural childbirth was more cost-effective for this population up to the percentage of 16% intrapartum C-section, considering willingness to pay of R$26,000. Above this percentage of intrapartum C-section, elective C-section would be the option of multiparous women with previous uterine scar.

In the probabilistic sensitivity analysis, elective C-section was cost-effective in 61% interactions for neonatal death avoided; in 54% for admission to neonatal ICU avoided; in 67% for uterine rupture avoided; in 66% for maternal morbidity avoided ( Figure 2 ), and in 57% for maternal death avoided, considering willingness to pay of R$26,000.

## DISCUSSION

Natural childbirth procedure has higher unit cost than that of elective C-section. However, in the analytical decision model, which included maternal and neonatal health outcomes, natural childbirth was more cost-effective than C-section for primiparous normal risk pregnant women. As C-section brings greater risk of admission to the ICU for the mother and the baby, the expected value is increased. Our findings reinforce the current ANS governmental policy that encourages natural childbirth for primiparous normal risk pregnant women ^c^ ^,^ ^k^ ^,^ ^o^ .

In contrast, for multiparous women with previous uterine scar, the cost-effectiveness analysis showed that repeat C-section is more cost-effective than natural childbirth after a C-section. However, the model presented was very sensitive to the parameter “probability of intrapartum C-section in the natural childbirth group”. If this probability is less than 16%, natural childbirth becomes cost-effective. In view of this result and the absence of similar analyzes in Brazil, more studies should evaluate the effectiveness of this procedure before adopting the absolute recommendation of C-sections. The risk of surgical complications is greater in places with deficient infrastructure or limited capacity to guarantee safe surgeries performance ^b^ .

Considering the limitations of this health care model for the promotion of natural childbirth, its review approaches at least two relevant aspects. The first is related to the arrangement that allows the availability of the professional in full-time, without impairing the development of other professional and personal activities. The arrangements around on-call shifts at the places of delivery may be one of the possibilities. Another central aspect is the inclusion of obstetric nursing in these teams, mainly considering the positive impact of their performance on the quality of care ^19^ .

The *Parto Adequado* project, which aims to identify innovative and viable models of attention to delivery and birth, presented results for the 26 hospitals participating in the first phase of the initiative. Among them, a 43% average increase of natural childbirth was verified. In 18 months, more than 10,000 C-sections without clinical indication were avoided in the hospitals participating in the initiative ^p^ . Private hospitals that adopted the model with on-call staff, collaboration between medical staff and obstetric nursing, and members of the Baby Friendly Hospital Initiative, also presented a lower rate of C-sections and better perinatal health outcomes.

Models of delivery and birth care in supplemental health other than those considered in this cost-effectiveness analysis could contribute to the performance of natural childbirth, also with impact on the cost of the procedure for the provider and the financer.

This is the first cost-effectiveness study from the perspective of supplemental health care in Brazil with this objective. In addition, the studies of this area in other countries refer to the public health system and not to the private component. Some studies have estimated the procedure cost for this perspective in Brazil ^21^ ^,^ ^22^ . However, the method used, the financing structure and the care organization are different, which makes it difficult to compare the results.

A study that analyzed the profile of deliveries performed by a small health care provider in Ceará, between 2011 and 2012, found average values that among providers varied from R$1,333.05 to R$1,876.20. However, the study does not detail the healthcare resources included in the invoice, or the type of delivery ^22^ . In a study carried out in Belo Horizonte, state Minas Gerais, health consequences and costs of C-section and natural childbirth were measured. In this research, 22% delivered pregnancies had C-section indication, and 72% of those pregnancies without C-section indication occurred by this way. C-section presented a cost slightly higher than R$77.00. If the effect of complications on costs was removed, C-section did not present higher costs than natural childbirth ^21^ .

This study has limitations. No national studies comparing the procedures studied for the population at normal risk were found. Thus, it was necessary to use the international literature available to identify the parameters used in the decision analysis model. The percentage of intrapartum C-section (24%) used for multiparous women influenced the results, making natural childbirth less cost-effective. Due to the impact of this variable, the sensitivity analysis considered that 8% women who went into labor would undergo intrapartum C-section according to data from the study “Birth in Brazil”. This percentage refers to any sample of this national survey, which included mostly primiparous pregnant women (79%) and, to a lesser extent, multiparous women with one or more scars ^18^ . The study did not present results for population subgroups, and using it for the reference case is not possible.

The cost data were based on the national health plan operators’ pricing tables. These tables are a reference for payment of providers, and their values vary greatly depending on the health care provider, its location, the negotiation with the hospitals, supply and medicine commercial brands, and each provider’s care routine.

Human resources remuneration was the greatest magnitude cost item in the study, and it was obtained from CBHPM table, a resolution of the Federal Council of Medicine, which adopts a minimum standard of remuneration for medical procedures with regard to Supplemental Health Care. In addition, the table does not have information on operating room rates and hospital daily rates, which are often negotiated separately between operators and providers. This is one of the limitations of this work, because we did not have information from all Brazilian regions, so that we could use a value that represented country’s reality. The health care providers used as a base act nationally, but the values we obtained refer to the Southeast region and Federal District.

Brazil does not have a cost-effectiveness threshold for SUS and for Supplemental Health Care as one of the criteria to subsidize health decision-making. We used the proposal of 71% GDP per capita for middle income countries, which referred to the outcome quality-adjusted life year (QALY) ^16^ . Therefore, we suggest caution when interpreting our results.

This study considered only full-term pregnancies and births. One of the discussions about scheduled C-sections is the birth of late preterm newborns (34–36 weeks of gestation). Thus, the percentage of admissions to neonatal ICU for C-section may be higher, considering the group of late preterm infants born from iatrogenic C-sections ^22^ ^,^ ^23^ .

This evaluation included immediate health outcomes that could occur within the time horizon studied. Important factors such as prevalence of breastfeeding, experience with childbirth, and pain and desire for new pregnancies should be discussed in the choice of the delivery type, and included as outcomes in future analyzes.

This study considered the perspective of supplemental health care from the perspective of the financer. Other perspectives should be considered, such as the perspective of hospitals as care providers. The role of obstetricians should be considered in studies of this nature due to their relevance to perinatal care, often determining the delivery type and the guidelines provided to pregnant women in prenatal care. The duration of each procedure is different, as the availability needed for each one. While a C-section can be scheduled on the best day and hour for the woman, natural childbirth is unexpected. It is necessary professional and bed availability at the maternity hospital. These issues may have less impact for health care providers, but they are issues to consider for hospitals and obstetricians that need to be available.

The reduction of C-section high rates in Brazil is a challenge, especially in supplemental health care, which has absolute predominance of surgical deliveries. The identification of C-sections without clinical justification, and the reason for their accomplishment, could contribute to understanding the high rates of supplemental health care, collaborating to new control and audit proposals.

## Footnotes

a Ministério da Saúde (BR), Departamento de Informática do SUS (DATASUS). Informações de saúde (TABNET): estatísticas vitais. Brasília (DF); s.d. [cited 2013 Aug 20]. Available from: http://www2.datasus.gov.br/DATASUS/index.php?area=0205

b Organização Mundial de Saúde, Departamento de Saúde Reprodutiva e Pesquisa. Declaração da OMS sobre Taxas de Cesáreas: resumo executivo. Genebra: OMS; 2014 [cited 2016 Oct 27]. Available from: http://apps.who.int/iris/bitstream/10665/161442/3/WHO_RHR_15.02_por.pdf

c Ministério da Saúde (BR), Secretaria de Ciência, Tecnologia e Insumos Estratégicos, Comissão Nacional de Incorporação de Tecnologias no SUS. Diretrizes de Atenção à Gestante: a operação cesariana. Brasília (DF); 2016 [cited 2016 Sep 16]. Protocolo Nº 19. Available from: http://conitec.gov.br/images/Consultas/Relatorios/2016/Relatorio_Diretrizes_Cesariana_N179.pdf

d Agência Nacional de Saúde Suplementar. Idoso na saúde suplementar: uma urgência para a saúde da sociedade e sustentabilidade do setor. Projeto idoso bem cuidado. Rio de Janeiro: ANS; 2016 [cited 2017 Apr 15]. Available from: http://www.ans.gov.br/images/stories/Materiais_para_pesquisa/Materiais_por_assunto/web_final_livro_idosos.pdf

e Agência Nacional de Saúde Suplementar. Perfil do Setor: dados gerais. Rio de Janeiro: ANS; s.d. [cited 2017 Apr 13]. Available from: http://www.ans.gov.br/perfil-do-setor/dados-gerais

f Agência Nacional de Saúde Suplementar. Atualização das taxas de partos na saúde suplementar. Rio de Janeiro: ANS; 2016 [cited 2016 Nov 3]. Available from: http://www.ans.gov.br/aans/noticias-ans/numeros-do-setor/3324-atualizacao-das-taxas-de-partos-na-saude-suplementar

g Ministério da Saúde (BR), Departamento de Informática do SUS (DATASUS). Sistema de Informações sobre Nascidos Vivos - SINASC. Brasília (DF) [cited 2014 Feb 12]. Available from: http://tabnet.datasus.gov.br/cgi/tabcgi.exe?sinasc/cnv/nvuf.def

n Câmara de Regulação do Mercado de Medicamentos, Conselho de Ministros. Resolução nº 3, de 4 de maio de 2009. Proíbe a aplicação de Preço Máximo ao Consumidor – PMC a medicamentos de uso restrito a hospitais. Brasília (DF); 2009 [cited 2016 Dec 27]. Available from: http://www.simpro.com.br/documentos/resolucao_03_09_restrito.pdf

o Ministério da Saúde (BR), Secretaria de Ciência, Tecnologia e Insumos Estratégicos, Comissão Nacional de Incorporação de Tecnologias no SUS. Diretriz Nacional de Assistência ao Parto Normal: relatório de recomendação. Brasília (DF); 2016 [cited 2017 Jan 25]. Available from: http://conitec.gov.br/images/Consultas/2016/Relatorio_Diretriz-PartoNormal_CP.pdf

p Agência Nacional de Saúde Suplmentar. Projeto Parto Adequado - Fase 2: inscrições prorrogadas. Roi de Janeiro: ANS; 2017 [cited 2017 Jan 30]. Available from: http://www.ans.gov.br/aans/noticias-ans/qualidade-da-saude/3682-projeto-parto-adequado-fase-2-ultimos-dias-para-inscricoes-2

h Agência Nacional de Saúde Suplementar. Taxas de partos cesáreos por operadora de plano de saúde. Rio de Janeiro: ANS; 2016 [cited 2016 Nov 3]. Available from: http://www.ans.gov.br/planos-de-saude-e-operadoras/informacoes-e-avaliacoes-de-operadoras/taxas-de-partos-cesareos-por-operadora-de-plano-de-saude

i Agência Nacional de Saúde Suplementar; Sociedade Beneficente Israelita Brasileira Hospital Albert Eistein; Institute for Healthcare Improvement. Cartilha nova organização do cuidado ao parto e nascimento para melhores resultados de saúde: Projeto Parto Adequado -Fase 1. Rio de Janeiro: ANS; 2016 [cited 2016 Nov 7]. Available from: http://www.ans.gov.br/images/stories/Materiais_para_pesquisa/Materiais_por_assunto/web_total_parto_adequado.pdf

j Agência Nacional de Saúde Suplementar. Resolução Normativa - RN Nº 368, de 6 de Janeiro de 2015. Rio de Janeiro: ANS; 2015 [cited 2016 Nov 7]. Available from: http://www.ans.gov.br/component/legislacao/?view=legislacao&task=TextoLei&format=raw&id=Mjg5Mg==

k Agência Nacional de Saúde Suplementar. Estímulo ao parto normal: organização da atenção ao pré-natal, parto e nascimento. Rio de Janeiro: ANS; s.d. [cited 2016 Dec 27]. Available from: http://www.ans.gov.br/images/stories/noticias/Parto_Adequado_final.pdf

l Ministério da Saúde (BR), Departamento de Informática do SUS (DATASUS). Internações hospitalares no SUS - por local de internação - Brasil. Brasília (DF) [cited 2014 Feb 3]. Available from: http://tabnet.datasus.gov.br/cgi/deftohtm.exe?sih/cnv/sxuf.def

m Associação Médica Brasileira. Classificação Brasileira Hierarquizada de Procedimentos Médicos – CBHPM – 2016. Barueri: Manole; 2016. [cited 2017 Apr 11]. Available from: https://amb.org.br/cbhpm/
